# To kill or not to kill: A systematic literature review of high-stakes moral decision-making measures and their psychometric properties

**DOI:** 10.3389/fpsyg.2022.1063607

**Published:** 2023-01-09

**Authors:** Benjamin Kai Ni, Bruce D. Burns, Karina K. L. Mak, Suncica Lah, Diego S. Silva, Micah B. Goldwater, Sabina Kleitman

**Affiliations:** ^1^School of Psychology, University of Sydney, Sydney, NSW, Australia; ^2^School of Public Health, University of Sydney, Sydney, NSW, Australia

**Keywords:** moral decision-making, moral reasoning, moral dilemma, individual difference, psychometrics, measurement

## Abstract

**Introduction:**

The present systematic review investigates the psychological tools available for capturing high-stakes decisions involving life-death content and their psychometric properties. Valid measurement of these individual differences will provide crucial information in the personnel selection and training in fields where high-stakes moral issues exist (e.g., military, medicine). To our knowledge, this is the first systematic examination of such instruments.

**Methods:**

Systematic searches of 6 electronic databases were conducted according to the PRISMA guidelines. An appraisal tool evaluated the quality of identified measures. Twenty studies met pre-determined inclusion criteria. Moral decision-making was assessed with either a self-report scale (*n* = 3) or moral dilemmas (*n* = 17).

**Results:**

The findings identified two measures, the Defining Issues Test and the Oxford Utilitarianism Scale as psychometrically sound measures of moral decision-making. However, they are unlikely to be considered “gold standard” measures due to their theoretically specific, but limited, scope. Overall, the findings suggest that research in the area has been scattered. There is a lack of consensus on the definition of moral decision-making, and a lack of cross-validation on how different measures of moral decision-making relate to each other. This presents a gap between theory and empirical measurement in moral decision-making. Further work is needed for a unified conceptualization of moral decision-making to pave the way to both theory development and the development of well-validated measurement tools, and this review provides a critical foundation for both.

## Introduction

Our world is full of volatile situations, including the current pandemic and recent wars, in which individuals must make high-stakes moral decisions. For example, in the first wave of the COVID-19 pandemic where medical staff and resources were overwhelmed, doctors and nurses were faced with moral decisions about whether to prioritize younger patients (who have a greater chance of survival) and whether to prohibit family visits to patients in ICU (Kuylen et al., 2021).

Given how important these consequences can be, it is important to ask if there are individual differences in moral decision-making in high-stakes situations which involve life-death decisions, and can we measure such differences? The existence of individual differences would imply that there are distinct and stable patterns in how people think, feel, and, importantly, act morally. Measuring individual differences would allow us to better understand, capture, and predict moral decision-making There are diverse government and private institutions that need to have established protocols of screening and selecting people who face high-stakes moral dilemmas (e.g., dealing with the sick, prisoners, victims of war) often in high-pressure situations. Having standardized and systematic information on measuring not moral reasoning, but moral decision-making is essential to generate and sustain trust in such organizations and to set the ground rules for their personnel. Thus, a systematic assessment of moral decision-making measures is vital as it will provide a much-needed foundation for screening and selection of personnel in fields where encounters with contentious high-stake moral issues are likely, such as military, medical, and legal professions.

### Moral decision-making: Concept and definition

Moral decision-making refers to any decisions made within the “moral domain”, including judgments, evaluations, and response choices (Smetana, 2006). However, the term “moral decision-making” has not always been used in past research, and instead terms such as “moral reasoning,” “moral judgment,” and “moral cognition” (Garrigan et al., 2018) have been used, sometimes interchangeably. Moral reasoning has also been defined as decision-making that includes moral and ethical components (Bucciarelli et al., 2008; Martí-Vilar et al., 2021). However, moral decision-making may not be dependent on reasoning and cognition alone (Richardson, 2018), rather emotion and intuition may also play key roles (Greene et al., 2001; Haidt, 2001). Therefore, we consider moral decision-making an umbrella term that encompasses reasoning, emotions, and intuitions regarding ethical and moral questions.

### Which is the focus, action, or actor?

Most contemporary research in moral decision-making has employed an act-based approach (Uhlmann et al., 2015). Specifically, researchers are interested in how individuals come to believe whether an *action* is morally right or wrong. A classic example is the Trolley Dilemma (Foot, 1967) where one must consider whether a trolley, which is on track to kill several people, should be actively diverted to another track where it will kill one person instead. Such sacrificial dilemmas have been adopted from philosophy and used to empirically probe what factors are taken into account in moral decision-making (see Christensen and Gomila, 2012 for a review). In the act-based approach it is the characteristics of the situation that are the focus of moral decision making.

In contrast to the act-based approach, recent research suggests that a *person*-centered approach may yield a better understanding of people's moral judgment. This approach focuses on individuals as the unit of analysis for moral evaluations rather than on acts (Uhlmann et al., 2015). This approach proposes that people are fundamentally motivated to acquire information about the moral character of others. Therefore, the features of an act that seem most informative of character often hold more weight than either the consequences of the act or whether a moral rule has been broken. Indeed, there is growing evidence to suggest that when faced with moral judgements people are focused on making inferences about moral character (Pizarro and Tannenbaum, 2012; Goodwin et al., 2014). Such moral character inferences are unlikely to be only a product of the features of the situation but also the traits of the maker of the inferences. This suggests that there could be robust individual differences in moral decision-making. That is, people make *systematic* choices about what is morally right or wrong despite varying situational factors. This paper systematically reviews evidence for individual differences in act-based moral research.

### Sources of individual difference in moral decision-making

There are two influential theories in moral psychology that may elucidate how individuals may differ in moral decision-making—Kohlberg's (1984) Moral Development Theory and Haidt's (2001) Social Intuitionist Theory. Given that there could be measures of individual differences in moral decision-making that are based on each of these theories, we should first describe them.

#### Moral development theory

Kohlberg's Moral Development Theory posits that moral development entails employing increasingly complex *cognitive* rationales for moral decision-making (Lapsley, 1992). The increasing complexity in *cognitive* processes is detailed in six qualitatively different stages, where moral development entails progression from the first to the last stage (Mathes, 2021).

In the first two stages, known as the pre-conventional stages, moral decision-making is concerned with instrumental purposes. In other words, individuals' moral behaviors in these stages are acted out for the purpose of avoiding punishment and obtaining pleasure (Mathes, 2021). In Stages 3 and 4, known as the conventional stages, the moral behaviors of individuals are concerned with social norms and conventions, as well as interpersonal (e.g., family and friends) and social (e.g., authority) approval (Blasi, 1990). In the last two stages, known as the post-conventional stages, moral decision-making is driven by clearly defined moral principles that are independent of the authority of groups holding these principles and one's identification with these groups (Ísaksson, 1979).

According to Moral Development Theory, individual differences in moral decision-making may arise from differences in moral development and maturity. However, there are several unresolved issues with this approach. First, the Moral Development Theory argues for a universal sequential trajectory of development. Attributing individual differences in moral decision-making merely to levels of moral maturity is almost certainly an oversimplification. Second, the theory's narrow focus on complex cognitive processes potentially excludes other factors (e.g., emotion, intuition) important to moral decision-making. Research on “moral dumbfounding” found that people can judge offensive yet harmless acts (e.g., incest with birth control) to be wrong but are unable to explain their reasoning or provide a justification (Haidt et al.).^1^ Therefore, Haidt et al. (see text footnote 1) argued that judgment in moral dumbfounding tasks is based on automatic and intuitive processes (e.g., feelings of rightness or wrongness). Thus, differences in moral decision-making between individuals cannot be explained by *cognitive* processes alone.

Despite these criticisms Kohlberg's theory has been influential on psychological research into moral decision making. As a result, there are measures of individual differences based on this theory that we expect to be part of this systematic review.

#### The social intuitionist model

The second influential theory in moral psychology is Haidt's (2001) Social Intuitionist Model. The Social Intuitionist Model argues against a rationalist model where moral judgments and decisions are reached through complex *cognitive* processes. Instead, Haidt (2001) argues that moral judgments and decisions are dependent primarily on one's moral *emotions* and *intuitions*. Moral reasoning, in his view, mostly serves as a *post-hoc* process to justify the established moral judgment.

The Social Intuitionist Model argues that moral intuition, much like language, evolved as a major adaptation for a social species while also requiring shaping from social and cultural institutions (Haidt, 2001). Therefore, it is both innate and enculturated. One's moral intuition can be considered a mixed product of innate predispositions (Fiske, 1991, 1992) and a unique developmental environment consisting of family, peers, and culture (Whiting and Child, 1953; Harris, 1995). Variation in people's moral intuitions may provide a basis for individual differences in moral decision-making that are not dependent on reasoning alone.

Emotions also play an important role for individuals in moral decision-making. Haidt (2003) discusses several families of emotions that are of relevance: other-condemning (contempt, anger, disgust), self-conscious (shame, embarrassment, guilt), other-suffering (sympathy, compassion), other-praising (gratitude, awe, elevation). Haidt (2003) argues that emotions place the person in a motivational and cognitive state in which there is an increased tendency to engage in actions that fulfill the emotion-related goals (e.g., revenge, comforting). Malti and Krettenauer (2013) conducted a meta-analysis and found that the ability to attribute emotion to moral actions (e.g., guilt over moral transgression, pride over prosocial actions) is linked to prosocial and antisocial behaviors among children and adolescents. Therefore, variability in the ability and tendencies for a broad range of moral emotions certainly has implications for moral decision-making. Thus, a comprehensive theory of the psychology of moral decision-making should bring these theories together by positing that moral decision-making is a broad construct that encompasses *reasoning, emotions*, and *intuitions*.

A comprehensive systematic literature review, using a standardized quality appraisal tool, is needed to apprehend and evaluate psychometric properties of the different high-stakes moral decision measures that are rooted in the different theories, aiming to clarify and possibly integrate them for future research. The findings will inform theories of moral decision-making, including their key models and definitions. We will first outline the findings from existing literature reviews, including their shortcomings. Second, we will define the key aims of this review. Third, we will situate different measures within their relevant frameworks while evaluating their psychometric properties, providing a key foundation for an informed assessment of their usefulness to capture high-stakes moral decision-making. We will then determine a gold standard measure of moral decision-making using the focus and definition proposed in this review.

### The present systematic review

Two influential psychological theories outlined the possibility that people can differ meaningfully in moral decision-making. The next step is to ask how empirical research has tried to measure these differences in moral decision-making.

#### Existing systematic reviews and meta-analyses

To date, two studies have systematically reviewed existing measures of moral decision-making. Villegas de Posada and Vargas-Trujillo's (2015) meta-analysis found that the development of moral reasoning positively correlated with domain-specific actions (real life, honesty, altruism, and resistance to conformity) and domain-general actions. Martí-Vilar et al. (2021) conducted a systematic review of existing moral reasoning measures and their reported psychometric properties. They identified 21 measures that could fall under one of four categories: (1) Kohlbergian Models, (2) Prosocial Moral Reasoning Models, (3) Moral Dilemmas, and (4) Other or Unspecified Models. While 21 measures were identified, only a few measures were represented in most of the studies examined and the rest received limited testing. The Defining Issues Test (Rest, 1974), based on the Kohlbergian Model, was one of the most commonly used measures. While these systematic reviews are informative of the current state of empirical research in moral decision-making, they have two shortcomings that the present review seeks to address. First, the quality of the evidence for the moral decision-making instruments' psychometric properties was not evaluated against pre-determined criteria. A standardized criteria framework for measuring the quality of evidence allows for a *systematic* examination of each measure's psychometric properties, as well as a comparison of their relative strengths. One measure may reliably measure a narrow aspect of moral decision-making, whereas another measure captures broader aspects but less reliably. These differences across measures can inform our selection of measures for different purposes.

Second, the studies considered were not only focused on high-stakes (life and death) situations. Instead, they allowed substantial variability in the context in which moral decisions were made (e.g., business, education, medicine, engineering, and science). However, this contextual variability may be problematic. The context often included domain-specific moral issues that are already addressed by guidelines and policies (e.g., there is a “correct” answer determined by an authority) and thus cannot capture meaningful differences in individual choices that do not follow prescribed rules. In contrast, measures of moral decision-making that involve life and death result in dilemmas where there is less consensus on what the right decision or judgment is. Importantly, determining whether systematic individual differences exist here can help us to understand, and predict, moral decision-making and behaviors, and thus aid development of theories of moral decision-making. From the applied perspective, this information provides a key platform for the screening and selection of personnel in various fields where people have to face high-stakes decisions, such as military, medical, and legal professions.

#### Systematic review: Aims

Therefore, the present systematic review aims to: (1) identify and examine existing measures of moral decision-making that involve life/death content when no clear and agreed rules exist; (2) evaluate the psychometric properties presented in construction and validation studies against a standardized quality appraisal tool (Terwee et al., 2007); (3) discuss the conceptualization of the construct and assess the usefulness of the identified measures; and (4) ascertain whether a gold standard measure of moral decision-making using the broad definition adopted in this review exists, and if not whether promising measures exist. The present review will follow the PRISMA Statement and guidelines for conducting and reporting systematic reviews (Liberati et al., 2009).

## Methods

### Search strategy

**Figure 1:**
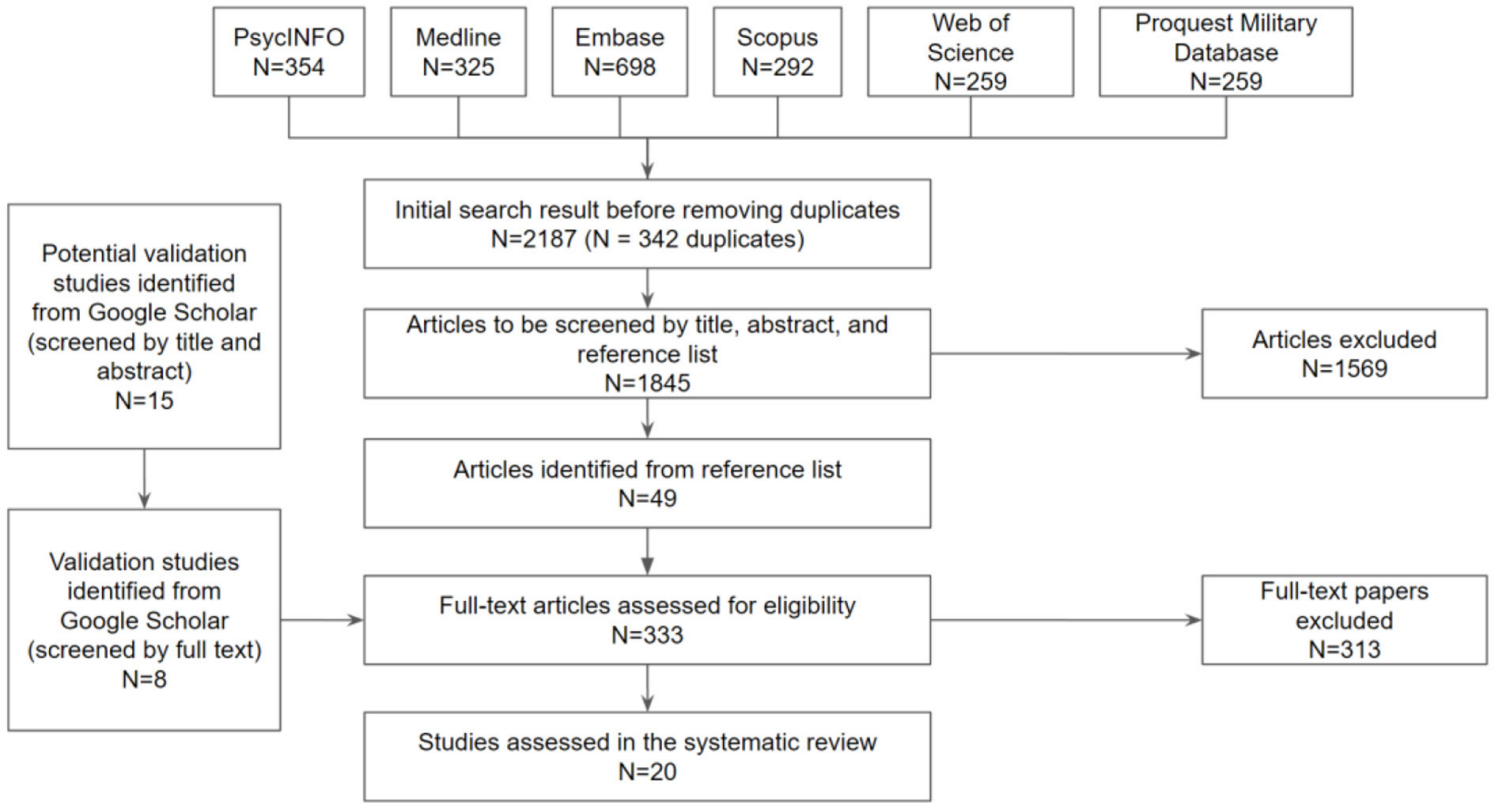
Flow diagram of the study selection process from systematic searches.

Electronic searches were conducted in six databases (see Figure 1): PsycINFO, Web of Science, Scopus, Medline, Embase, and the ProQuest Military Database. These databases were selected based on the focus of this systematic literature review on life/death content, thus we included medical and military databases in addition to the three more general scientific databases. The final search was conducted in all databases on 13th May 2021. Relevant studies were identified using a combination of keywords. PsycINFO, Medline, and Embase also allow searching by subject headings, which are subsequently used to attain additional papers not captured by keyword searches. Generally, the search strategy aims to identify an intersection of studies that focused on (1) moral decision-making, and (2) measurement.

Reference lists of all included studies were also manually screened for potentially relevant publications. Additionally, potential validation studies were searched by manually screening studies that cited the original measure construction studies on Google Scholar. The search for additional validation studies through Google Scholar was conducted between 14th July 2021 and 29th July 2021.

### Inclusion criteria

Peer-reviewed journal articles, book chapters, and unpublished dissertations were included in the review if they were an original quantitative research study that developed and/or validated a measure of moral decision-making. Studies were included if their aims were to develop or validate a measure of moral decision-making: (1) contains life/death content, or (2) includes sacrificial moral dilemma(s). Studies were included if the sample consisted of at least 50% adults (i.e., over 18 years of age). Thus, some studies that tested high school students were also included, but this characteristic was recorded. Studies published in the English language were included, regardless of whether the study used a non-English speaking sample, but this characteristic was also recorded.

### Exclusion criteria

Studies were excluded if more than 50% of the sample were not adults, were a from non-peer-reviewed journal, conference proceedings, non-empirical studies, or were not written in the English language. Studies were excluded if the measures of moral decision-making: (1) did not contain life/death measures, or (2) did not include sacrificial moral dilemma(s).

### Selection process

The entire selection process was conducted by BN and KM authors. Search results were initially screened by title and abstract to exclude studies that did not meet the inclusion criteria. For the remaining papers, full-text papers were obtained and evaluated in accordance with the inclusion/exclusion criteria.

### Data extraction and quality assessment

The psychometric properties of all measures in the included studies were assessed using a published quality appraisal tool (Terwee et al., 2007) developed to assess the quality of health status questionnaires' validity, reliability, and responsiveness. Although moral decision-making is not in the domain of health status, the quality appraisal tool has been used in reviews that assessed the psychometric properties of individual difference measures (e.g., imposter phenomenon; Mak et al., 2019). Therefore, this measurement framework was considered an appropriate tool for evaluating studies that examined the psychometric properties of moral decision-making measures.

The appraisal framework evaluates nine properties: (1) content validity, (2) internal consistency, (3) criterion validity, (4) construct validity, (5) reproducibility-agreement, (6) reproducibility-reliability, (7) responsiveness, (8) floor or ceiling effects, and (9) interpretability. The definitions and criteria of quality for each psychometric property are displayed in Table 1. Similar to Mak et al.'s (2019) study, certain criteria from the original framework were amended due to the nature of the moral decision-making measures. These amendments were noted in Table 1. For example, item selection, a criterion of content validity, should only be applied to the original test construction studies and not follow-up validation studies.

**Table 1:** Summary of search terms from six databases.

**Database**	**Search terms**
PsycINFO	((Decision Making **AND** (Morality **OR** Ethics)) **OR** “moral decision making” “ethical decision making” **OR** “moral reasoning”) **AND** (*Measurement*^a^ OR “moral dilemma”)
Embase^b^	(*Decision-Making* **AND** (*Morals* **OR** *Ethics*)) **AND** (“moral decision making” **OR** “ethical decision making” **OR** “moral reasoning”)
Medline^c^	(*Decision-Making* **AND** (*Morals* **OR** *Ethics*)) **AND** (“moral decision making” **OR** “ethical decision making” **OR** “moral reasoning”)
Web of science	(“ethical decision making” **OR** “moral decision making” **OR** “moral reasoning”) **AND** (measurement **OR** psychometr*OR “moral dilemma”)
Scopus	(“ethical decision making” **OR** “moral decision making” **OR** “moral reasoning”) **AND** (measurement **OR** psychometr*OR “moral dilemma”)
Proquest military database	(“ethical decision making” **OR** “moral decision making” **OR** “moral reasoning”) **AND** (measurement **OR** psychometr*OR “moral dilemma”)

Subject headings are in *italics*. Keywords are in quotation marks (“”).

^a^Related subheadings for measurement are also selected to include more relevant results. For the full syntax of search terms (see Appendix A).

^b, c^Embase and Medline do not have “Measurement” as a subject heading. Therefore, searches of subject headings and keywords were solely focused on moral decision-making. Relevant papers are screened and selected manually.

Each assessed criterion received a rating score of “+” as good, “?” as intermediately rated, “–” as negatively rated, or a “0” if no information was provided on that criterion for a specific study. An “N/A” (not applicable) rating was assigned for a particular criterion if it is impossible to evaluate the criterion due to the research design used in the study. For example, responsiveness is a criterion assessing how well the measure detects clinically important changes over time, which is not applicable to studies that are non-longitudinal.

The two researchers (BN and KM) independently evaluated each included study and evaluated their psychometric properties against the amended quality framework. Discrepancies in scoring were discussed at calibration meetings to arrive at a consensus.

## Results

The initial search returned 2,187 results (including 342 duplicates) but after screening, most excluded because the study: (1) was not a validation study, (2) the measure used was qualitative or not life-or-death related, or (3) not published in the English language. The flow diagram in Figure 1 documents the review process.

Overall, we were left with 20 studies to fully evaluate. The identified measures in these studies generally adopted one of two formats: moral dilemmas or self-report scales. Moral dilemma measures were used in 16 included studies. The main moral dilemma measures identified included: (1) the Defining Issues Test (Rest, 1974) and its revised versions, (2) measures using the Process Dissociation (PD) Model (Conway and Gawronski, 2013), and (3) measures using the Consequences, Norms, Inaction (CNI) Model (Gawronski et al., 2017). Three self-report measures were identified in the remaining three studies. Table 2 describes the included studies organized by the type of measurement and ascending year of publication within the same group.

**Table 2:** Adapted criteria for quality of psychometric properties and scoring system (Terwee et al., 2007).

	**Property**	**Definition**		**Quality criteria**	**Criteria amendment**
1	Content validity	The extent to which the domain of interest is comprehensively sampled by the items in the questionnaire	+	A clear description is provided of the measurement aim, the target population, the concepts that are being measured, and the item selection **AND** target population and (investigators OR experts) were involved in item selection;	(1) Target population - clear description of the **sample characteristics** (e.g., undergraduate students, M and SD and/or range of age, gender). (2) Item selection should be theoretically driven - only relevant to test construction papers.
			?	A clear description of above-mentioned aspects is lacking **OR** only target population involved **OR** doubtful design or method;	
			−	No target population involvement;	
			0	No information found on target population involvement.	
2	Internal consistency	The extent to which items in a (sub)scale are intercorrelated, thus measuring the same construct	+	Factor analyses performed on adequate sample size (7 * # items and ≥100) AND Cronbach's alpha(s) calculated per dimension AND Cronbach's alpha(s) between 0.70 and 0.95;	
			?	No factor analysis OR doubtful design or method;	
			−	Cronbach's alpha(s) < 0.70 or >0.95, despite adequate design and method;	
			0	No information found on internal consistency.	
3	Criterion validity	The extent to which scores on a particular questionnaire relate to a gold standard	+	Convincing arguments that gold standard is “gold” AND correlation with gold standard ≥ 0.70;	
			?	No convincing arguments that gold standard is “gold” OR doubtful design or method;	
			−	Correlation with gold standard < 0.70, despite adequate design and method;	
			0	No information found on criterion validity.	There is a gold standard that the researchers haven't referred to
			N/A		No gold standard mentioned
4	Construct validity	The extent to which scores on a particular questionnaire relate to other measures in a manner that is consistent with theoretically derived hypotheses concerning the concepts that are being measured	+	Specific hypotheses were formulated AND at least 75% of the results are in accordance with these hypotheses;	(1) Main hypothesis confirmed. (2) 50% instead of 75% are in accordance with these hypotheses. (3) Hypotheses should be about proposed relationships between the measure and other theoretically related constructs or about proposed group differences as opposed to hypothesized factor structure. (4) Statements of examining convergent and divergent validity are sufficient to be considered as hypotheses when assessing construct validity.
			?	Doubtful design or method (e.g., no hypotheses);	
			−	Less than 75% of hypotheses were confirmed, despite adequate design and methods;	
			0	No information found on construct validity.	No hypotheses or exploratory hypotheses only.
5	Reproducibility: agreement	The extent to which the scores on repeated measures are close to each other (absolute measurement error)	+	MIC < SDC OR MIC outside the LOA OR convincing arguments that agreement is acceptable;	
			?	Doubtful design or method OR (MIC not defined AND no convincing arguments that agreement is acceptable);	
			−	MIC ≥ SDC OR MIC equals or inside LOA, despite adequate design and method;	
			0	No information found on agreement.	
			N/A		Study is non-longitudinal.
6	Reproducibility: reliability	The extent to which patients^a^ can be distinguished from each other, despite measurement errors (relative measurement error)	+	ICC or weighted Kappa ≥ 0.70;	
			?	Doubtful design or method (e.g., time interval not mentioned);	
			−	ICC or weighted Kappa < 0.70, despite adequate design and method;	
			0	No information found on reliability.	
			N/A		Study is non-longitudinal.
7	Responsiveness	The ability of a questionnaire to detect clinically^a^ important changes over time	+	SDC or SDC < MIC OR MIC outside the LOA OR RR > 1.96 OR AUC ≥ 0.70;	
			?	Doubtful design or method;	
			−	SDC or SDC ≥ MIC OR MIC equals or inside LOA OR RR ≤ 1.96 OR AUC < 0.70, despite adequate design and methods;	
			0	No information found on responsiveness.	
			N/A		Study is non-longitudinal.
8	Floor and ceiling effects	The number of respondents who achieved the lowest or highest possible score	+	≤ 15% of the respondents achieved the highest or lowest possible scores;	
			?	Doubtful design or method;	
			−	>15% of the respondents achieved the highest or lowest possible scores, despite adequate design and methods;	
			0	No information found on interpretation.	
9	Interpretability	The degree to which one can assign qualitative meaning to quantitative scores	+	Mean and SD scores presented of at least four relevant subgroups of patients and MIC defined;	Relevant subgroups = groups that differ in meaningful ways (e.g., demographics, between-subjects experimental manipulation).
			?	Doubtful design or method OR less than four subgroups OR no MIC defined;	Incomplete presentation of means and SD scores
			0	No information found on interpretation.	

Adapted with permission from “Impostor phenomenon measurement scales: a systematic review”, by Mak et al. (2019).

^a^These terms are used in Terwee et al. (2007) but were interpreted more broadly here.

*M*, mean; SD, standard deviation; MIC, minimal important change (smallest difference in score in the domain of interest which patients perceive as beneficial and would agree to, in the absence of side effects and excessive costs); SDC, smallest detectable change (smallest within person change, above measurement error. A positive is given when SDC or the limits of agreement are smaller than the MIC); LOA, limits of agreement; ICC, intraclass correlation; RR, responsiveness ratio; AUC, area under the curve; +, Positive rating; ?, Intermediate rating; −, Negative rating; 0, No information provided; N/A, Not applicable.

It is important to notice that there is substantial variability in what the moral decision-making measures aim to measure and their theoretical basis. This implies that there is a lack of consensus regarding the construct of moral decision-making, and therefore each measure may only capture some of its dimensions. The next section gives a brief description of the identified measures.

### Measures based on moral dilemmas

#### The Defining Issues Test and revised versions

The Defining Issues Test (DIT; Rest, 1974) and its revised versions, the DIT-2 (Rest et al., 1999), and the behavioral Defining Issues Test (bDIT; Choi et al., 2019), were used in seven studies. The Defining Issues Test was based on Kohlberg's (1984) Moral Development Theory. The DIT consists of six sacrificial dilemma stories. After each story, the participant is given a list of reasons for (e.g., sacrificing a life to save more lives) or against an action (e.g., not killing anyone even if it saves others) and asked to rank and rate the importance of each reason. These reasons stem from Kohlberg's stages (2–6) of moral reasoning and can be grouped into three categories: personal interests (Stage 2), maintaining social norms (Stages 3 and 4), or post-conventional perspectives (Stages 5A, 5B, and 6). The DIT quantifies a person's moral development by their likelihood of endorsing post-conventional reasons (i.e., the P score). While the DIT produces several scores representing reliance on each stage of moral development (i.e., stage scores from stages 2–6), the P score is the most widely used index (Rest et al., 1999).

The DIT-2 contains five of the six dilemmas from the original DIT with updated language and generates the N2 instead of P score. The N2 score, like the P score, considers the preference for post-conventional reasoning. In addition, the N2 score takes into account the disagreement with less sophisticated schemas (Rest et al., 1999).

The bDIT contains three dilemmas and measures behavioral responses, such as reaction time (Choi et al., 2019). Instead of rating the importance of reasons for action/inaction, participants were given a limited amount of time to select one of the three presented behavioral responses. These three responses represent the three moral schemas: Personal Interest, Maintaining Norms, and Post-Conventional Reasoning.

#### Measures using the Process Dissociation Model

A set of moral dilemmas that evaluates a person's inclinations for utilitarianism and deontology separately was created by Conway and Gawronski (2013). This set of moral dilemmas was based on the Process Dissociation (PD) Model (Jacoby, 1991). Psychologists typically define utilitarianism as the principle whereby the morality of an action is determined by its consequences (Conway and Gawronski, 2013). On the other hand, deontology is defined as the principle that the morality of an action is determined by its intrinsic nature (e.g., causing harm is wrong regardless of the consequences). While earlier sacrificial dilemmas pit utilitarianism against deontology (e.g., Greene et al., 2001), Conway and Gawronski (2013) argued that the endorsement of one does not necessarily imply a rejection of the other. Therefore, participants' ratings of the appropriateness of action in 20 dilemmas were analyzed using process dissociation to extract inclinations toward both utilitarianism and deontology. Jang (2020) translated the PD into Korean and conducted a study to validate the measure.

#### Measures using the CNI model

The CNI model (Gawronski et al., 2017) further developed the Process Dissociation Model by addressing another problem with the traditional approach. In addition to the two inclinations underlined by the Process Dissociation Model, Gawronski et al. (2017) argued that there is a third component, a general tendency for inaction, that may play a role in moral decision-making. In a morally ambiguous situation, a person may prefer to not act because they do not want to inject themselves into events, rather than due to a strong inclination toward deontology or utilitarianism. In traditional moral dilemmas, the action always leads to sacrificial killing, which conflates with a preference for inaction. Using the multinomial processing tree method, Gawronski et al. (2017) developed the CNI model, which is a new set of 24 dilemmas that measured participants' sensitivity to **C**onsequences (inclination for utilitarianism in the Process Dissociation Model), sensitivity to **N**orms (inclination for deontology in the Process Dissociation Model), and a general tendency for Inaction. Körner et al. (2020) expanded the battery from 24 to 48 to improve its suitability in individual difference research.

#### Other moral dilemmas

The remaining moral dilemma studies each identified one measure. Bore's (2001) Morality of Justice and Care (MOJAC) scale conceptualized moral dilemmas as the conflict between the rights of the individual (e.g., stealing a drug to save one's sick wife) and the rights of the collective (e.g., stealing is wrong).

Christensen et al. (2014) systematically developed a battery of moral dilemmas based on four conceptually meaningful factors: personal force, benefit recipient, evitability, and intentionality. Additionally, contextual factors such as the word count, framing, situational antecedents, number of individuals involved, types of trade-off (e.g., killing vs. stealing, lying), and whether your action will be known to others, were controlled for. Christensen et al. (2014) were interested in whether their conceptual factors influenced participants' decisions, arousal, valence, and reaction times.

Fleischhut et al. (2017) investigated the effect of hindsight in moral decision-making. They were interested in how moral decisions are influenced if participants had information on their actions' consequences. Fleischhut et al. (2017) generated dilemmas in which actions to avert negative outcomes had probable side effects, and then created three information conditions. In the *foresight* condition, participants were provided with no further information and asked for a decision. In the *hindsight-good* and *hindsight-bad* conditions, participants were given additional information stating that the negative side effects either occurred (*bad* condition) or did not occur (*good* condition). Participants were asked to judge the permissibility of the action and the probability of the negative side effect occurring in the future.

Kimhi (2014) developed moral dilemmas in war-related scenarios (e.g., whether to open fire on the enemy at the risk of harming civilians). Participants' decisions, their perceived appropriateness, confidence, the difficulty of their decisions, and the estimated probability of specific outcomes (e.g., civilians being killed) were measured.

Lotto et al. (2014) investigated the effects of intention and self-involvement in moral decision-making. They constructed 75 moral dilemmas consisting of 30 “instrumental dilemmas,” 30 “incidental dilemmas,” and 15 fillers. Instrumental dilemmas described killing an individual as a means to save others (e.g., killing and taking an innocent person's organs to treat five patients in need of transplants). On the other hand, incidental dilemmas described killing an individual as a foreseen but unintended consequence (e.g., switching the trolley onto another track where there is another worker). Additionally, approximately half of the dilemmas in each condition were self-involved (i.e., killing saves one's own life and others), and half were other-involved (i.e., killing saves others only). Lotto et al. (2014) were interested in the effects of intention and self-involvement on participants' decisions, their rating of an action's moral acceptability, and their affective reactions.

Carmona-Perera et al. (2013) translated and adapted the moral dilemmas from Greene et al. (2001). The moral dilemmas were adapted to investigate brain activities when participants were dealing with morally conflicting situations. The battery of dilemmas consisted of three groups: non-moral stories, moral-impersonal stories (e.g., flipping a switch to divert the trolley from killing five workers), and moral-personal stories (e.g., pushing a man off the bridge to stop the trolley from killing five workers). More personal moral dilemmas were expected to be more conflicting and associated with both less willingness to take action and heightened brain activity. The personal dilemmas were further divided into high-conflict (dilemmas that had low consensus on the appropriate decision in previous studies) and low-conflict (dilemmas that had high consensus). Carmona-Perera et al. (2013) were interested in the decisions participants would make, the difficulty they felt when making the decision, and the proportion of congruent decisions as an index of rationality (e.g., saying no to risky investment decisions).

### Description of self-report scales

#### ABB scale

The ABB scale, named after the initials of the authors—Abdellaoui et al. (2016)—was created to measure people's judgments on personal, conventional, and moral transgressions. For each type of transgression, four scenarios were given and participants rated how serious and how defensible the action is, and whether the transgressor should be rejected.

#### Oxford Utilitarianism Scale

Kahane et al.'s (2018) Oxford Utilitarianism Scale aimed to measure two aspects of utilitarianism. The first aspect, instrumental harm, measures whether individuals find causing harm permissible if it leads to more moral good overall. The second aspect, impartial beneficence, assesses whether individuals maximize overall moral goodness even if it conflicts with self-interest (e.g., donating one's majority of income to charity). Kahane et al. (2018) argued that existing moral dilemma measures predominantly focused on *instrumental harm* but overlooked *impartial beneficence*. To address this gap, the Oxford Utilitarianism Scale measures both of these factors.

#### Punishment Orientation Questionnaire

The Punishment Orientation Questionnaire (POQ; Yamamoto and Maeder, 2019) aimed to measure what principles people engage with when thinking about punishment. The POQ captures two general principles that underlie the motivations behind punishment—utilitarianism (i.e., deterrence of future transgression) and retributivism (i.e., an eye for an eye). Furthermore, each principle is divided into a Prohibitive dimension and a Permissive dimension, resulting in four subscales: (1) Prohibitive Utilitarianism (limiting punishment based on utility), (2) Prohibitive Retributivism (aversion to punishing if it means hurting innocent people), (3) Permissive Utilitarianism (willingness to give harsh punishment based on the benefits thereof), and (4) Permissive Retributivism (desire for just desserts).

### Assessment of psychometric properties

The assessment of psychometric properties was conducted in accordance with the amended version of the quality appraisal framework defined by Terwee et al. (2007). Two reviewers independently rated each included study against the nine psychometric properties of the appraisal framework (Terwee et al., 2007). Agreement between the two reviewers on the criteria of adequacy was 87.78% and this equates to a Kappa of *k* = 0.87. Kappa is an inter-rater agreement statistic that controls for the agreement expected based on chance alone and a Kappa of 0.87 represents a substantial degree of agreement between raters (Cohen, 1960). Table 3 presents detailed information on the measures' factorial structure, reliability estimates, and findings in relation to other variables or group differences.

**Table 3:** Included study descriptions.

**References**	**Measures**	**Study type**	**Statistical analysis**	**Number of scenarios/items**	**Questions asked**	**Study population**	**Age (mean)**	**Sex ratio^a^**
**Moral dilemmas**								
**DIT**								
Martin et al. (1977)		Validation	ANOVA	Six scenarios	Importance on 12 items for each scenario	Sample 1: 60 junior high school, sample 2: 200 high school students, sample 3: 105 college students	Sample 1: 13.9 years, sample 2: 17.3 years, sample 3: 20.2 years	Sample 1: 33 males, 27 females, sample 2: 93 males, 107 females, sample 4: 34 males, 71 females
Davison and Robbins (1978)		Validation	Cronbach's α, test-retest reliability, *t*-test, correlation	Six scenarios	Importance on 12 items for each scenario	1,703 from six samples including high school students, undergraduate and graduate students, and adults	Ranged 15–82 years	Most samples reported to have approximately even split between males and females.
**DIT-2**								
Rest et al. (1999)		Adaptation	ANOVA, correlation, Cronbach's α, regression, *t*-test	Five scenarios	Importance on 12 items for each scenario	Sample 1: 47 ninth-grade students, sample 2: 35 senior high graduates, new freshmen, sample 3: 65 college seniors, sample 4: 53 graduate school and professional school students	Sample 1: 14.64 years, SD = 0.53, sample 2: 18.51 years, SD = 2.03, sample 3: 21.55 years, SD = 3.11, sample 4: 29.06 years, SD = 5.90	Sample 1: 34% female, sample 2: 77% female, sample 3: 77% female, sample 4: 45% female
Mitchell (2000)		Validation	ANOVA, factor analysis, reliability, ANCOVA, correlation	Five scenarios	Importance on 12 items for each scenario	1,534 consisted of 26 samples collected by a third-party research center from 1998 to 1999.		606 males, 904 females
Mayhew et al. (2015)		Validation	Regression	Five scenarios	Action, 12-item scale	923 (first-year undergraduates in the US)	Not reported	38.4% male, 61.6% female
Choi et al. (2020)		Validation	CFA	Five scenarios	Importance on 12 items for each scenario	39,409 (US citizens in university, collected by a third-party research center between 2000 and 2009)	Ranged 17–26 years	21,139 males (47.2%), 23,272 females (52%)
**bDIT**								
Choi et al. (2019)		Adaptation	Reliability (tetrachoric correlation), differential item functioning analysis, logistic regression, ANOVA	Three scenarios	Behavioral decision and eight questions asking the rationale behind decision for each story	353 (introductory psychology students in the US)	18.64 years, SD = 1.20	81 males, 271 females
**Process Dissociation Model**								
Conway and Gawronski (2013)		Construction	S1: *t*-test, correlation, regression, S2: *t*-test, correlation, ANOVA, S3: *t*-test, ANOVA	20 scenarios	S1: appropriateness, S2: appropriateness, S3: appropriateness	S1: 112 (undergraduate students), S2: 57 (undergraduate students), S3: 275 (MTurk)	S1: 19.23 years, SD = 5.20, S2: 18.37 years, SD = 0.96, S3: 34.08 years, SD = 11.73	S1: 30 males, 82 females, S2: 28 males, 29 females, S3: 118 males, 156 females
Jang (2020)		Validation	Correlation, Mann-Whitney test	20 scenarios	Appropriateness, probability of taking action, how happy	465 (Korean adults)	31.37 years, SD = 14.20	163 males, 300 females
**CNI model**								
Gawronski et al. (2017)		Construction	S1: *t*-test, *g*-test, S2: *t*-test, *g*-test, S3: *t*-test, *g*-test, S4: *t*-test, *g*-test	24 scenarios	S1: acceptability, S2: acceptability, S3: acceptability, action, S4: acceptability	S1a: 201 (MTurk), S1b: 197 (MTurk), S2a: 194 (MTurk), S2b: 194 (MTurk), S3a: 186 (MTurk), S3b: 189 (MTurk), S4a: 184 (MTurk), S4b: 198 (MTurk)	S1a: 32.20 years, SD = 10.96, S1b: 35.77 years, SD = 11.47, S2a: 34.26 years, SD = 11.90, S2b: 36.36 years, SD = 12.40, S3a: 35.77 years, SD = 12.79, S3b: 34.72 years, SD = 10.69, S4a: not reported, S4b: not reported	S1a: 106 males, 95 females, S1b: 95 males, 102 females, S2a: 96 males, 97 females, S2b: 103 males, 91 females, S3a: 86 males, 100 females, S3b: 91 males, 98 females, S4a: not reported, S4b: not reported
Körner et al. (2020)		Adaptation, validation	S1: correlation, S2: correlation	48 scenarios	S1: acceptability, S2: action	S1a: 161 (MTurk), S1b: 177 (MTurk), S2a: 196 (MTurk), S2b: 189 (MTurk)	S1a: 37 years, SD = 11, S1b: 33 years, SD = 9, S2a: 35 years, SD = 10, S2b: 34 years, SD = 9	S1a: 84 males, 72 females, S1b: 105 males, 65 females, S2a: 93 males, 102 females, S2b: 96 males, 90 females
**Other moral dilemmas**								
Bore (2001)		Construction, adaptation, validation	S1: Cronbach's α, PCA, correlation, S2: Cronbach's α, PCA, correlation, S3: Cronbach's α, PCA, correlation, S4: Cronbach's α, *t*-test, correlation, second-order factor analysis (varimax rotation), S5: Cronbach's α, *t*-test, ANOVA, correlation, test-retest reliability, PCA, S6: *t*-test, S7: Cronbach's α, ANOVA, S8a: Cronbach's α, *t*-test, regression, ANOVA, S8b: Interview (non-quantitative), S9: correlation, regression (stepwise), PCA	S1: 35 items (three scenarios), S2: 45 items (four scenarios), S3-6: 24 items (three scenarios), S7: 24 items, except for the New Zealand samples, which completed the 45-item version, S8a: 45 items, S8b: 45 items	Decision items (e.g., action should be taken/is prohibited/is not important)	S1: 882 (Medical school applicants), S2: 2,906 (Medical school applicants), S3: 2,862 (Medial school applicants), S4: 84 (first year psychology students), 82 (Medical school applicants), S5: 232 (Bachelor of Medicine students), S6: 16 (ethical clinicians), S7: 2,862 (sample from S3), 113 (New Zealand medical students, sample A), 123 (New Zealand medical students, sample B), 360 (Israel medical school applicants, sample A), 626 (Israel medical school applicants, sample B), 67 (Fiji medical students), S8a: 58 (Medical school applicants), S8b: 45 (sample from S8a), S9: 113 (Medical students, sample A), 123 (Medical students, sample B)	S1: 19.3 years, SD = 3.9, S2: 18.6 years, SD = 3.3, S3: 19.9 years, SD = 4.8, S4: 22.5 years, SD = 8.2, S5: 23.6 years, SD = 5.0, S6: Not reported, S7: 19.9 years, SD = 4.8 (sample from S3), 20.2 years, SD = 3.0 (New Zealand sample A), 19.9 years, SD = 2.4 (New Zealand sample B), 22.7 years, SD = 3.7 (Israel sample A), 22.5 years, SD = 2.5 (Israel sample B), 19.0 years, SD = 2.3 (Fiji sample), S8a: not reported, S8b: not reported, S9: 20.2 years, SD = 3.0 (sample A), 19.9 years, SD = 2.4 (sample B)	S1: 368 males, 510 females, S2: 1,267 males, 1,634 females, S3: 1,334 males, 1,525 females, S4: 42 males, 121 females, S5: 104 males, 128 females, S6: Not reported, S7: 1,334 males, 1,525 females (sample from S3), 50 males, 62 females (New Zealand sample A), 44 males, 79 females (New Zealand sample B), 185 males, 174 females (Israel sample A), 294 males, 323 females (Israel sample B), 29 males, 33 females (Fiji sample), S8a: 31 males, 27 females, S8b: 25 males, 20 females, S9: 50 males, 62 females (sample A), 44 males, 78 females (sample B)
Carmona-Perera et al. (2013)		Translation, adaptation, validation	ANOVA	60 scenarios	Action, difficulty, congruency.	154 (Spanish undergraduates)	21.51 years, ranged 18–54 years	29 males, 120 females
Christensen et al. (2014)		Adaptation, validation	S1: ANOVA, *t*-test, regression, S2: ANOVA, *t*-test	46 scenarios	Rate level of arousal, rate perceived valence of the dilemma (S1) additional question re: action (S2)	S1: 62 (undergraduate psychology students), S2: 43 (undergraduate psychology students)	S1: 21.0 years, SD = 5.35 S2: 20.65, SD = 5.52	S1: 19 males, 43 females, S2: 13 males, 30 females
Kimhi (2014)		Construction	Correlation, path analysis	One scenario	Reaction (Y/N) to seven possibilities of action, rate level of confidence on 5pt likert scale to each decision	346 Israeli Defense Force soldiers (202 regular and 144 active reserve)	22.50 years	Not reported
Lotto et al. (2014)		Construction	ANOVA	75 scenarios	Action	120 (University students)	19.96 years, SD = 2.70	55 males, 65 females
Fleischhut et al. (2017)		Construction	ANOVA, correlations	Six scenarios	Appropriateness, moral permissibility, probability of outcomes, probability estimate, rank importance	731 (MTurk)	32.6 years, SD = 12.1	405 males, 326 females
**Self-report scales**								
Abdellaoui et al. (2016)	ABB	Construction	S1: ANOVA, PCA, correlation, S2: ANOVA	12 item scale		S1: 521, S2: 193 (prison inmates)	S1: 28.1 years, SD = 7.4, S2: 23.72 years	S1: 58.93% male, 41.97 female, S2: 193 males (100%)
Kahane et al. (2018)	OUS	Construction, validation	S1: EFA and CFA, S2: CFA, correlation, S3: *t*-test, correlation	Nine item scale		S1: 960 (MTurk), S2: 282 (MTurk), S3: 81 (experts in Moral Philosophy)	S1: 35 years, SD = 12.11, S2: 39 years, SD = 12.66, S3: 32 years, SD = 9.72	S1: 489 females, S2: 178 females, S3: 23 females
Yamamoto and Maeder (2019)	POQ	Construction, validation	S1: EFA, IRT, S2: CFA, S3: correlations, regression	17 item scale		S1: 199 (MTurk), S2: 188 (MTurk), S3: 179 (MTurk)	S1: 33.9 years, SD = 10.7, S2: 32.8 years, SD = 10.6, S3: 36 years, SD = 10.3	S1: 69 males, 120 females, S2: Not reported, S3: 95 males, 84 females

^a^There are missing values of sex/gender in some studies. Therefore, numbers reported in Sex Ratio do not necessarily add up to total sample size.

bDIT, behavioral Defining Issues Test; DIT, Defining Issues Test; DIT-2, Defining Issues Test Version 2; MD, moral dilemma; MOJAC, Moral Orientation of Justice and Care Scale; PMC, Professional Moral Courage Scale; POQ, punishment orientation questionnaire; OUS, The Oxford Utilitarianism Scale; ABB, the scale of social and moral judgments (named after the initials of the authors); URPO, utilitarian and retributive punishment orientation; ANOVA, analysis of variance; DIF, differential item functioning analysis; ANCOVA, analysis of covariance; CFA, confirmatory factor analysis; EFA, exploratory factor analysis; IRT, item response theory; PAF, principle axis factoring.

The ratings of psychometric properties for each study are reported in Table 4. None of the studies reported information on floor and ceiling effects. Therefore, all studies were assigned a score of “0” for no information reported on floor and ceiling effects. The ratings of psychometric properties in Terwee et al.'s (2007) framework are reported in Table 5.

**Table 4:** Summary of factorial structure, reliability estimates, and relationships of the measure with other variables/group differences.

**Study**	**Factorial structure and reliability**	**Relationship with other variables/group differences**			
**DIT**					
Martin et al. (1977)	Kirstof's reliability (0.70)	College > high school > junior high			
Davison and Robbins (1978)	Cronbach's α (0.77–0.82), test-retest (0.71–0.81; 2–4 years)	Cognitive ability (*r* = 0.43^**^), comprehension of moral issues (*r* = 0.65^**^), law and order orientation (*r* = −0.50^**^), political tolerance (*r* = 0.50^**^)			
**DIT-2**					
Rest et al. (1999)	Cronbach's α (0.81)	DIT P score (*r* = 0.71^**^), education level (*r* = 0.69^**^), age (*r* = 0.56^**^), attitudes toward human rights (higher scores indicate greater advocacy for civil liberties, *r* = 0.50^**^)			
Mitchell (2000)	Three-factor solution (personal interest, maintaining norms, post-conventional), Cronbach's α's (0.727 for N2 score, 0.619 for *P* score)	Higher education > lower education, liberal > conservative, women > men			
Mayhew et al. (2015)		Took DIT-2 three times > took DIT-2 two times			
Choi et al. (2020)	Bi-factor model with a general factor G and 3 lower-order factors (personal interest, maintaining norms, post-conventional), Cronbach's alpha (0.840)				
**bDIT**					
Choi et al. (2019)	Tetrachoric correlation (0.74)	DIT (*r* = 0.71)			
**Process Dissociation Model**					
Conway and Gawronski (2013)		**Deontological** inclination:		**Utilitarian** inclination:	
		Utilitarian inclination (*r* = 0.09), empathic concern (*r* = 0.28^**^), perspective-taking (*r* = 0.32^**^), religiosity (*r* = 0.26^**^), moral identity internalization (*r* = 0.22^*^)		Need for cognition (*r* = 0.18), moral identity internalization (*r* = 0.23^**^), high cognitive load < low cognitive load	
Jang (2020)		**Deontological** inclination		**Utilitarian** inclination	
		Utilitarian inclination (*r* = −0.23^**^), Oxford Utilitarianism Scale (*r* = −0.27^**^), antisocial personality disorder (*r* = −0.13^**^), antisocial tendencies (*r* = −0.17^**^), older < younger		Oxford Utilitarianism Scale (*r* = 0.18^**^), women < men, higher education < lower education	
**CNI model**					
Gawronski et al. (2017)		Sensitivity to **consequences**	Sensitivity to **norms**	Tendency for **inaction**	
		High psychopathy < low psychopathy	Women > men, high psychopathy < low psychopathy	Women > men, high cognitive load > low cognitive load, action > judgment, high psychopathy < low psychopathy	
Körner et al. (2020)			Sensitivity to **consequences**	Sensitivity to **norms**	Tendency for **inaction**
		Psychopathy	*r* = −0.194^**^ to −0.357^**^	*r* = −0.494^**^ to −0.613^**^	*r* = −0.143 to −0.299^**^
		Empathic concern	*r* = −0.051 to 0.144	*r* = 0.175^*^ to 0.384^**^	*r* = −0.023 to 0.164^*^
		Need for cognition	*r* = 0.022 to 0.166^*^	*r* = 0.077 to 0.270^**^	*r* = 0.027 to 0.112
		Impartial beneficence	*r* = −0.078 to −0.202^**^	*r* = −0.172^*^ to −0.348^**^	*r* = −0.010 to −0.287^**^
		Instrumental harm	*r* = −0.029 to −0.142	*r* = −0.411^**^ to −0.561^**^	*r* = −0.145^*^ to −0.239^**^
		Behavioral inhibition	*r* = 0.032 to 0.157	*r* = 0.053 to 0.167^*^	*r* = −0.038 to 0.098
		Behavioral activation	*r* = 0.004 to −0.279^**^	*r* = −0.050 to −0.149^*^	*r* = −0.040 to −0.180^*^
		Moral identity internalization	*r* = 0.107 to 0.199^**^	*r* = 0.347^**^ to 0.466^**^	*r* = 0.087 to 0.238^**^
		religiosity	*r* = −0.146^*^ to −0.350^**^	*r* = 0.101 to −0.235^**^	*r* = −0.181^*^ to 0.011
**Other moral dilemmas**					
Bore (2001)	One-factor solution from PCA, Cronbach's α: 0.88 (35-item), 0.90 (45 item), 0.83 (24-item), Test-retest (0.77, 1 year)	Age (*r* = 0.2^**^ to 0.23^**^), gender (*r* = 0.08^*^ to 0.14^**^), DIT P score (−0.10^*^), DIT decision (0.51^**^), narcissism (*r* = −0.15^**^ to −0.16^**^), aloofness (*r* = −0.04 to −0.15^**^),		Confidence (*r* = 0.10^*^ to 0.19^**^), empathy (*r* = −0.01 to 0.00), power (*r* = −0.19^*^), hedonism (*r* = −0.27^**^), benevolence (*r* = 0.20^*^), conformity (*r* = 0.30^**^)	
Carmona-Perera et al. (2013)	Cronbach's α (0.705)	Affirmative decisions: non-moral > moral impersonal > moral personal, high-conflict > low-conflict, perceived difficulty: moral personal > moral impersonal & non-moral, high-conflict > low-conflict, congruent answers: non-moral > moral impersonal & moral personal			
Christensen et al. (2014)		Arousal: personal force > impersonal force, self-beneficial > other-beneficial, empathy (*r* = 0.289^*^), valence: personal force (more negative), self-beneficial (more negative), personal force × intentionality (accidental harm was rated more negative than instrumental harm in impersonal force condition), benefit recipient × intentionality (accidental harm was rated as more negative than instrumental harm in self-beneficial condition)		Reaction Time: personal < impersonal, self-beneficial < other-beneficial, arousal (*r* = −0.434^**^), EMPATHY (*r* = −0.325^*^)	
Kimhi (2014)	Cronbach's α's: 0.70 (decision) 0.74 (confidence) 0.80 (difficulty)	Compared to regular soldiers, reserve soldiers are more likely to take action (*r* = −0.141^**^), feel more confident (*r* = 0.107^*^) and less difficult (*r* = −0.102) about the decision, and more likely to be left-wing oriented (*r* = −0.262^**^). Left-wing political orientation was associated with less likelihood of action (*r* = −0.264^**^) and higher perceived difficulty about the decision (*r* = 0.211^**^).			
Lotto et al. (2014)		Affirmative decisions: incidental killing > instrumental killing, self-beneficial > other-beneficial, decision times: incidental killing > instrumental killing, acceptability ratings: incidental killing > instrumental killing, self-beneficial < other-beneficial, valence ratings: self-beneficial (more unpleasant), women (more unpleasant)		Arousal ratings: incidental killing > instrumental killing, self-beneficial > other-beneficial, women > men	
Fleischhut et al. (2017)		Impermissibility rating: foresight (no info on consequence) & hindsight-bad (side effects of action occurred) > hindsight-good (side effects of action did not occur) estimated likelihood of side effects occurring: foresight & hindsight-bad > hindsight-good participants who judged action as impermissible > participants who judged action as permissible (*r* = −0.39)			
**Self-report scales**					
Abdellaoui et al. (2016)	Three-factor solution, Cronbach's α:0.88 (overall), 0.78 (moral values), 0.82 (conventional values), 0.80 (personal values)	Seriousness: inmates < control defensibility/excusability: inmates < control tolerance (seeing transgression as serious but more defensible and not rejecting the transgressor): inmates < control			
Kahane et al. (2018)	Two-factor solution, Cronbach's α:0.81 (impartial beneficence), 0.79 (instrumental harm)	Impartial beneficence: instrumental harm (*r* = 0.14^*^), explicit utilitarianism (*r* = 0.37^*^), classic sacrificial dilemmas (*r* = −0.21^**^), greater good dilemmas (*r* = 0.50^**^), empathic concern (*r* = 0.33^**^), identification with all of humanity (*r* = 0.33^**^), hypothetical donation (*r* = 0.40^**^), environmental protection (*r* = 0.14^**^), religiosity (*r* = 0.15^**^)		Instrumental harm: explicit utilitarianism (*r* = 0.13^*^), classic sacrificial dilemmas (*r* = −0.32^**^), greater good dilemmas (*r* = 0.07^**^), empathic concern (*r* = −0.16^**^), identification with all of humanity (*r* = −0.19^**^), environmental protection (*r* = −0.21^**^), economic conservatism (*r* = 0.18^**^), social conservatism (*r* = 0.18^**^)	
Yamamoto and Maeder (2019)	Four-factor solution, Cronbach's α: 0.84 (permissive retributive), 0.85 (permissive utilitarian), 0.79 (prohibitive retributive), 0.80 (prohibitive utilitarian)	Permissive retributive: future-time orientation (*r* = 0.23^*^), positive affect (*r* = 0.36^**^), death penalty for retribution (*r* = 0.55^**^), death penalty for deterrence (*r* = 0.49^**^)	Permissive utilitarian: past-time orientation (*r* = 0.21^**^), positive affect (*r* = 0.30^**^), death penalty for retribution (*r* = 0.57^**^), death penalty for deterrence (*r* = 0.64^**^), death qualification^a^ (*r* = −0.39^**^)	Prohibitive retributive: positive affect (*r* = −0.28^**^), negative affect (*r* = −0.15^*^), death penalty for retribution (*r* = −0.39^**^), death penalty for deterrence (*r* = −0.52^**^)	Prohibitive utilitarian: positive affect (*r* = −0.16^**^), death penalty for retribution (*r* = −0.27^**^), death penalty for deterrence (*r* = −0.38^**^), death qualification (*r* = 0.30^**^)

^a^Death qualification is a question asking participants whether their beliefs about the death penalty will impair their performance of their duties as jurors; higher scores indicate a response that prior beliefs about the death penalty do not interfere with performance of duty as a juror.

^*^p ≤ 0.05,

^**^p ≤ 0.01.

**Table 5:** Overview of scoring of psychometric properties in the included studies.

**Measures**	**Content validity**	**Internal consistency**	**Criterion validity**	**Construct validity**	**Reproducibility: agreement**	**Reproducibility: reliability**	**Responsiveness**	**Floor and ceiling effects**	**Interpretability**
**Moral dilemmas**									
**DIT**									
Martin et al. (1977)	+	?	N/A	0	N/A	N/A	N/A	0	?
Davison and Robbins (1978)	+	?	N/A	0	0	0	0	0	0
**DIT-2**									
Rest et al. (1999)	+	?	+	+	N/A	N/A	N/A	0	+
Mitchell (2000)	+	?	N/A	+	N/A	N/A	N/A	0	?
Mayhew et al. (2015)	+	0	N/A	0	0	0	0	0	0
Choi et al. (2020)	+	+	N/A	0	N/A	N/A	N/A	0	0
**bDIT**									
Choi et al. (2019)	+	?	+	0	N/A	N/A	N/A	0	0
**Process Dissociation Model**									
Conway and Gawronski (2013)	+	N/A	N/A	+	N/A	N/A	N/A	0	0
Jang (2020)	+	N/A	N/A	+	N/A	N/A	N/A	0	?
**CNI model**									
Gawronski et al. (2017)	+	N/A	N/A	+	N/A	N/A	N/A	0	0
Körner et al. (2020)	+	N/A	N/A	+	N/A	N/A	N/A	0	0
**Other moral dilemmas**									
Bore (2001)	+	+	N/A	?	0	0	0	0	+
Carmona-Perera et al. (2013)	+	?	N/A	0	N/A	N/A	N/A	0	0
Christensen et al. (2014)	+	0	N/A	0	N/A	N/A	N/A	0	?
Kimhi (2014)	+	?	N/A	+	N/A	N/A	N/A	0	0
Lotto et al. (2014)	+	0	N/A	0	N/A	N/A	N/A	0	?
Fleischhut et al. (2017)	+	0	N/A	0	N/A	N/A	N/A	0	?
**Self-report scales**									
Abdellaoui et al. (2016)	+	+	N/A	0	N/A	N/A	N/A	0	?
Kahane et al. (2018)	+	+	N/A	+	N/A	N/A	N/A	0	?
Yamamoto and Maeder (2019)	+	+	N/A	+	N/A	N/A	N/A	0	0

DIT, Defining Issues Test; DIT-2, Defining Issues Test Version 2; bDIT, behavioral Defining Issues Test; N/A, not applicable; +, Positive rating; ?, Intermediate rating; −, Negative rating; 0, No information provided.

### DIT and revised versions

Seven studies used the Defining Issues Test and its revised versions. For ***content*** ***validity***, all seven studies received positive ratings for content validity for providing adequate evidence on measurement aim, target population, and concepts being measured.

For ***internal consistency***, one study received a positive rating for internal consistency. Choi et al. (2020) conducted factor analyses on the DIT-2 (Cronbach's α = 0.84). Five studies reported Cronbach's α ranging between 0.70 and 0.82 but did not perform factor analyses. These studies were assigned intermediate ratings for internal consistency. Mayhew et al. (2015) was assigned “0” for internal consistency as no information on internal consistency was reported.

For ***criterion validity***, two studies developed revised versions of existing measures and used the original version as a benchmark for validation. Rest et al.'s (1999) DIT-2 (*r* = 0.71) and Choi et al.'s (2019) bDIT (*r* = 0.71) were validated against the DIT and were thus assigned positive ratings for criterion validity. The remaining studies did not mention a “gold standard” of moral decision-making measure. Therefore, they were assigned “N/A” for criterion validity.

For ***construct validity***, of the seven studies that investigated various versions of the DIT, two studies were assigned positive ratings for construct validity. Rest et al. (1999) hypothesized positive correlations between DIT-2 scores and age, education level, and attitudes toward human rights. Mitchell (2000) hypothesized positive correlations between DIT-2 scores and age and political liberalism. The remaining five studies were each assigned a score of “0” because they did not propose theoretically-driven hypotheses. Choi et al. (2020) investigated the factorial structure of the DIT-2. While specific hypotheses were proposed, they were neither about relations to other measures nor expected group differences. Therefore, a “0” was assigned for construct validity for no information on appropriate hypotheses.

***Reproducibility (agreement and reliability)*** and ***responsiveness*** are criteria that apply to repeated measures designs only. Agreement is defined as the extent to which scores on repeated measures are close to each other (absolute measurement error) (Terwee et al., 2007). Reliability (test-retest) is defined as the extent to which participants can be distinguished from each other, despite measurement error (Terwee et al., 2007). Responsiveness is the measure's ability to detect clinically important changes over time, however small the changes are.

Five studies did not examine repeated measures of moral decision-making, therefore these criteria were not applicable for these studies. These studies were assigned an “N/A” rating on Agreement, Reliability, and Responsiveness.

Of the remaining two studies that used repeated measures designs, neither referred to the required indices of Agreement (e.g., Minimal Important Change). Therefore, these studies were assigned a “0” score for no information provided on Agreement. For Reliability, neither study referred to the required indices of Reliability (e.g., Intraclass Correlation Coefficient, weighted Cohen's Kappa). Therefore, these studies were assigned a “0” score for no information provided. Nonetheless, these studies reported test-retest reliabilities, which are presented in Table 3.

Neither study referred to the required indices of Responsiveness (e.g., Smallest Detectable Change, Minimal Important Change, Guyatt's Responsiveness Ratio). Therefore, these studies were assigned a “0” score for no information provided.

***Interpretability*** is defined as the ability to assign qualitative meaning to quantitative scores (Terwee et al., 2007). Interpretability is important for health measures because it is crucial that the scores from the instrument reflect meaningful differences between groups (e.g., patient vs. control, gender, age). A positive rating for interpretability was given only if the study reported means and standard deviations of the measure for at least four subgroups. An intermediate score was given if there was incomplete reporting of statistics and/or less than four subgroups.

Rest et al. (1999) received a positive rating for interpretability for reporting means and standard deviations of the DIT-2 N2 scores for participants in four education levels (from ninth grade students to graduate and professional school students).

Two studies received intermediate ratings for interpretability. Martin et al. (1977) reported means (but not standard deviation) of DIT P scores among junior high school, senior high school, and college students. Mitchell (2000) reported means (but not standard deviations) of DIT-2 P and N2 scores among five groups of political identities (from very liberal to very conservative).

The remaining four studies were assigned a score of “0” indicating no information on interpretability was reported. This was either due to not having subgroups or not reporting descriptive statistics of the measure across the subgroups.

### Measures using the Process Dissociation Model

Two studies used PD measures. For ***content validity***, both studies were positively rated for providing adequate evidence on measurement aim, target population, and concepts being measured. As the construction study of the PD measure, Conway and Gawronski (2013) were theoretically driven in their item selection and thus received a positive rating for content validity.

For ***internal consistency***, both studies received “N/A” due to how the construct scores were calculated. The PD model takes responses from all dilemmas to produce a single score for each factor (i.e., propensities for utilitarian/deontological principles). This is contrary to self-report scales where multiple items measure the same construct such that analysis of internal consistency can be performed. Therefore, an “N/A” was assigned to these studies as the criteria for internal consistency was not applicable.

For ***criterion validity*, **neither study mentioned a “gold standard” of moral decision-making measure. Therefore, they were assigned “N/A” for criterion validity.

For ***construct validity***, the PD measures received one positive rating and one “0” score. Conway and Gawronski (2013) proposed specific hypotheses and found that deontological inclinations were positively correlated with empathic concern, perspective-taking, religiosity, and moral identity internalization. Jang (2020) translated and validated the Korean version of the PD measure. A score of “0” was assigned because the analyses were exploratory, and no hypotheses were proposed a priori.

For ***reproducibility (agreement and reliability) and responsiveness***, neither studies used a repeated measures design. Therefore, both studies were assigned an “N/A” rating.

For ***interpretability***, the PD measure received one intermediate rating and one “0” score. Jang (2020) reported means (but not standard deviation) of utilitarian and deontological inclinations of males and females. Conway and Gawronski (2013) did not report descriptive statistics for any subgroups.

### Measures using the CNI model

Two studies used measures adopting the CNI model. For ***content validity***, both studies were positively rated for providing adequate evidence on measurement aim, target population, and concepts being measured. As the construction study of the CNI measure, Gawronski et al. (2017) were theoretically driven in their item selection and thus received a positive rating for content validity.

For ***internal consistency***, similar to the PD measures, both studies received “N/A” ratings. The CNI model takes responses from all dilemmas to produce a single score for each factor (i.e., sensitivity to consequences, sensitivity to norms, general tendency for inaction). Therefore, the criteria for internal consistency was not applicable.

For ***criterion validity*, **neither study mentioned a “gold standard” of moral decision-making measure. Therefore, they were assigned “N/A” for criterion validity.

For ***construct validity***, both studies received positive ratings. Although Gawronski et al. (2017) and Körner et al. (2020) did not propose specific hypotheses regarding the model, each exploratory study (e.g., Studies 1a, 2a, 3a, 4a) was accompanied by a replication study (e.g., Studies 1b, 2b, 3b, 4b) that found supporting evidence for exploratory findings. We deemed this method to be appropriate for minimizing the risk of bias from retrospective explanations and evaluated the percentage of hypotheses supported based on the proportion of relationships replicated in the second study compared to the first study.

For ***reproducibility (agreement and reliability) and responsiveness***, neither study used a repeated measures design. Therefore, both studies were assigned an “N/A” rating.

For ***interpretability***, the CNI measure received two “0” scores. Neither study reported descriptive statistics for any subgroups.

### Other moral dilemmas

There are six studies that each identified one unique measure of moral decision-making. For ***content validity***, all studies received positive ratings for providing adequate evidence on measurement aim, target population, and concepts being measured. For construction studies, Bore (2001), Kimhi (2014), Lotto et al. (2014), and Fleischhut et al. (2017) were theoretically driven in their item selection and thus received a positive rating for content validity.

For ***internal consistency***, Bore (2001) received a positive rating for conducting factor analyses and reporting internal consistency estimates (Cronbach's α = 0.83–0.90). Carmona-Perera et al. (2013) and Kimhi (2014) were intermediately rated for reporting adequate internal consistency estimates (Cronbach's α > 0.70). Christensen et al. (2014), Lotto et al. (2014), and Fleischhut et al. (2017) did not report any reliability measures and were thus rated “0” for internal consistency.

For ***criterion validity***, none of the studies mentioned a “gold standard” of moral decision-making measure. Therefore, they were assigned “N/A” for criterion validity.

For ***construct validity***, Kimhi (2014) received a positive rating for formulating theoretically driven hypotheses and obtaining supporting evidence. Bore's (2001) MOJAC scale was intermediately rated for construct validity. This was because a number of hypothesized relationships (e.g., Right Wing Authoritarianism, emotional intelligence) were not supported. Christensen et al. (2014), Lotto et al. (2014), and Abdellaoui et al. (2016) did not propose specific hypotheses and were thus each assigned a score of “0”. Fleischhut et al. (2017) proposed specific hypotheses. However, a score of “0” was assigned because the hypothesized relationships were about group differences from experimental manipulations rather than theoretical relationships of the measure with other constructs.

For ***reproducibility (agreement and reliability) and responsiveness*, **only Bore (2001) used a repeated measures design. However, the studies did not refer to indices of agreement, reliability, or responsiveness (e.g., Minimal Important Change). Therefore, these studies were assigned a “0” score for no information provided on these criteria. The remaining five studies were rated “N/A” as the criteria are not applicable.

For ***interpretability***, Bore (2001) received a positive rating for reporting means and standard deviations of the MOJAC scale by groups based on the language spoken at home, years enrolled into medical school and cultural backgrounds. Christensen et al. (2014), Lotto et al. (2014), and Fleischhut et al. (2017) were intermediately rated either for reporting means but not standard deviations of the subgroups' scores, or not systematically reporting descriptive statistics of all subgroups. Carmona-Perera et al. (2013) and Kimhi (2014) were given “0” as there was no information on descriptive statistics of subgroups.

### Self-report scales

Three self-report scales were identified in three construction studies—the ABB Scale (Abdellaoui et al., 2016), the Oxford Utilitarianism Scale (Kahane et al., 2018), and the Punishment Orientation Questionnaire (Yamamoto and Maeder, 2019).

All three studies received positive ratings for ***content validity*** for providing adequate evidence on measurement aim, target population, and concepts being measured. Additionally, they were all theoretically driven in the construction and selection of scale items.

For ***internal consistency***, all three studies were positively rated for conducting factor analyses and reporting internal consistency estimates (Cronbach's α = 0.79–0.88).

For ***criterion validity*, **none of the studies mentioned a “gold standard” of moral decision-making measure. Therefore, they were assigned “N/A” for criterion validity.

For ***construct validity***, Kahane et al. (2018) and Yamamoto and Maeder (2019) received positive ratings for formulating theoretically driven hypotheses and obtaining supporting evidence. Abdellaoui et al. (2016) did not propose specific hypotheses and was thus assigned a score of “0”.

For ***reproducibility (agreement and reliability) and responsiveness***, none of the studies used a repeated measures design. Therefore, all three studies were assigned an “N/A” rating.

For ***interpretability***, two studies were intermediately rated. Abdellaoui et al. (2016) reported means (but not standard deviations) of seriousness and defensibility ratings of violations between prison inmates and the general population, between sex offenders and other offenders, and between recidivists and first-time offenders. Kahane et al. (2018) reported means and standard deviations of Instrumental Harm and Impartial Beneficence between self-identified Republicans and Democrats. Additionally, means and standard deviations of Instrumental Harm (but not Impartial Beneficence) were reported between men and women. Yamamoto was assigned a score of “0” due to not having subgroups.

## Discussion

The present systematic review had four aims: (1) identify existing measures of moral decision-making with life/death content, (2) evaluate the psychometric properties of these measures against a quality appraisal tool, (3) discuss the conceptualization of the construct and assess the usefulness of the identified measures, and (4) ascertain whether a gold standard measure of moral decision-making using the broad definition adopted in this review exists, and if not, whether promising measures exist. Below we will assess our degree of success in achieving each aim.

### Aims 1 and 2: Identifying and evaluating measures of moral decision-making

This review was successful in identifying twelve unique measures of moral decision-making in high-stakes situations. Nine of these were moral dilemma sets, of which only three were reported in more than one study (Defining Issues Test, Process Dissociation Model, and CNI Model), and the other three were self-report scales (ABB Scale, Oxford Utilitarianism Scale, Punishment Orientation Questionnaire).

#### Defining Issues Test and revised versions

Consistent with previous findings (Villegas de Posada and Vargas-Trujillo, 2015; Martí-Vilar et al., 2021), the Defining Issues Test (Rest, 1974) and its revised versions were the most commonly used measure in the identified studies. The DIT is one of the earliest measures developed to examine moral decision-making. The results of this systematic review found adequate evidence on factorial structure, internal consistency, and temporal stability. Moreover, the DIT has been validated against theoretically related constructs (e.g., cognitive ability, education, political orientation) and other measures of moral decision-making (e.g., Christensen et al., 2014). Ample evidence of reliability and validity suggests that the DIT and its revised versions (i.e., DIT-2, bDIT) may be a candidate for a gold standard for measuring moral decision-making. However, a limitation of the DIT is that it is based on Kohlberg's theory of moral reasoning, which is developmental in nature and, thus, the scope of the measure may be theoretically limited. Specifically, the difference in scores on the DIT may be attributed to the levels of moral reasoning development, and thus limits its usefulness for examining differences that may arise among morally mature people (e.g., utilitarian vs. deontological inclinations).

#### Process Dissociation Model and CNI model

The Process Dissociation Model was proposed to challenge the dichotomy between utilitarian and deontological tendencies that underlies earlier measures of moral decision-making (Conway and Gawronski, 2013). Rather than considering them as opposites, utilitarian and deontological tendencies are proposed as separate constructs. Given this theoretical framework, the PD measure was validated against theoretically related constructs (e.g., empathic concern, perspective-taking, religiosity, moral identity internalization) as well as the Oxford Utilitarianism Scale (Kahane et al., 2018). However, there is limited evidence on the reliability, especially internal and temporal, of the measure. Given its different scoring methods, traditional measures of internal consistency (e.g., Cronbach's α) are not applicable. Additionally, a lack of longitudinal studies means that there is no information on the temporal stability of these constructs. Such psychometric properties need to be examined for the measure to be considered a gold standard for measuring moral decision-making.

The CNI Model extended the PD Model by introducing a third factor—a general tendency for inaction. In morally ambiguous situations, people may prefer inaction over any choice of action. The CNI measure was validated against theoretically related constructs (e.g., psychopathy, empathic concern, need for cognition, behavioral activation/inhibition, moral identity internalization, and religiosity) and the Oxford Utilitarianism Scale (Kahane et al., 2018). However, the CNI Model shares the same limitation as the PD Model in the lack of evidence on reliability. Future studies will need to examine the internal consistency and temporal stability of the measure.

#### Single studies using moral dilemmas

Six other individual studies using different sets of moral dilemmas involving life and death scenarios were identified in this review. Five of these were intended to measure the effect of experimental manipulations (e.g., amount of information participants had before making a decision). Such measures were not intended for capturing robust individual differences, instead they focused on the state rather than trait aspects. Therefore, the evidence of their psychometric properties will not be discussed.

In the sixth, Bore (2001) developed the MOJAC scale to measure an individual's inclination toward the rights of the individual vs. the rights of the collective. The MOJAC scale demonstrated good internal consistency and test-retest reliability. Although some hypotheses were not supported, the MOJAC scale was related to some important theoretically relevant constructs (e.g., Power, Hedonism, Benevolence, and Conformity) and the DIT (Rest, 1974). Overall, the MOJAC scale appears to be a good individual differences measure of certain facets of moral decision-making. Its theoretical scope may need to be extended as moral decision-making in high-stakes situations goes beyond consideration of the rights of the individual versus those of the collective.

#### Self-report measures

The ABB scale (Abdellaoui et al., 2016) was intended to measure an individual's judgment of seriousness, defensibility, and tolerability of three types of transgressions—personal, conventional, and moral. While the scale has good internal consistency, it was not validated against any theoretically related constructs. The incomplete evidence of psychometric properties needs to be addressed for the measure to be used in research.

The Oxford Utilitarianism Scale (Kahane et al., 2018) is a self-report measure of utilitarianism in high-resolution by focusing on the two underlying factors—impartial beneficence and instrumental harm. The authors provided adequate evidence on factorial structure, internal consistency, and construct validity on the OUS. Furthermore, it has been evaluated against the Process Dissociation Model (Jang, 2020) and the CNI Model (Körner et al., 2020). However, in return for depth, the OUS has sacrificed its breadth in measurement. By focusing on utilitarianism, it overlooks other important factors such as the intentions of actions, motivations to conform to norms, and tendencies to avoid moral issues. Additionally, there is no information on the temporal stability of the constructs measured by the OUS.

The Punishment Orientation Questionnaire (Yamamoto and Maeder, 2019) looked at moral decision-making in the form of punishments. The scale measures two motivations behind punishment—utilitarianism and retributivism, each of which was further divided into prohibitive motivation and permissive motivation. There is good evidence of the factorial structure, internal consistency, and construct validity of the measure. However, the limited scope of POQ means that it is unlikely to be considered a gold standard measure, as moral decision-making entails more than just punishment. Moreover, the temporal stability of the punishment construct is yet to be examined.

### Aim 3: Conceptualization

The result of the present review indicates that a diverse set of theoretical frameworks has been used to conceptualize moral decision-making. Most notably, the DIT (Rest, 1974) and its revised versions were based on Kohlberg's (1984) Moral Development Theory. Almost all measures involved measuring utilitarianism–the OUS (Impartial Beneficence vs. Instrumental Harm), the POQ (Utilitarianism vs. Retributivism), the PD and CNI Models (Utilitarianism/Consequences, Deontology/Norms, and Inaction), and the MOJAC scale (Individual Rights vs. Collective Rights). However, none of the conceptualizations are broad enough to be considered moral decision-making. Instead, each conceptualization focuses on a sub-facet of moral decision-making. This creates a critical obstacle to the integration of the theory of moral decision-making in general, and limits the cross-validation of different measures against each other and other key measures in the nomological network. For instance, only two studies received a positive rating for criterion validity. The DIT-2 (Rest et al., 1999) and the bDIT (Choi et al., 2019) were both strongly correlated to the original DIT (Rest, 1974). All other studies were rated “N/A” indicating that it was impossible to evaluate criterion validity. Therefore, there is a lack of consensus on what models and theories should be the basis of understanding moral decision-making.

The remaining measures were traditional sacrificial dilemmas that pitted utilitarian decisions against deontological decisions (Carmona-Perera et al., 2013; Christensen et al., 2014; Kimhi, 2014; Lotto et al., 2014; Fleischhut et al., 2017) and were not intended to measure systematic individual differences in the way people approach and process moral decisions. Moreover, the breadth of the moral decision-making construct has not been captured by traditional sacrificial dilemmas. Although emotion and intuition play important roles in theoretical approaches to moral decision-making, such as Greene et al. (2001) and Haidt (2001), emotions were not measured in the scales we reviewed, and only once did we find a measure that the authors tried to validate against any aspect of emotion. Recent theories and empirical evidence suggest that cognitive processes may not be the only (or even the most important) factor in moral decision-making (Haidt, 2001). Moreover, the literature on how metacognitive processes (thinking about thinking; Flavell, 1979) are involved in moral decision-making is scarce. However, the emerging metacognitive Meta-reasoning model (Ackerman and Thompson, 2017) outlines processes that monitor the progress of our problem-solving and reasoning that foster an individual to take a particular action, and these constructs and processes are of direct relevance to moral decision-making. Therefore, the present review identifies a need for a more holistic approach that captures *broad and systematic individual differences in terms of both the breadth of scope and the systematic tendencies (e.g., trait-like factors) underlying moral judgements and their respective nomological network*. Studies that used sacrificial dilemmas were primarily interested in measuring the effects of experimentally manipulating contextual variables (e.g., number of lives saved, whether oneself benefits from sacrificial killing). The considerable influence of this experimental paradigm in the moral decision-making field may partly explain the lack of consensus and systematic conceptualization of moral decision-making, which affects investigations into this construct, its measurement models, and its relationship with other measures. Striving toward a consensus on models and theories is critical and necessary for advancing research in moral decision-making.

### Aim 4: Toward a gold standard

The final aim of the present review was to identify a gold standard measure of moral decision-making. We do not believe we have identified such a measure, but our review highlights what is needed. Two identified measures—the DIT (and its revised versions) and the OUS—seem promising given their psychometric soundness, however the DIT relies on a particular conceptualization of moral decision-making and the OUS only aims to measure one aspect. A gold standard would require agreement as to what a moral decision-making scale should measure, but at the moment there is a scattered conceptualization of moral decision-making across different measures. Therefore, the DIT and the OUS may serve as gold standards of what they aim to measure, as long as researchers are aware of their theoretically limited scope. If we are to have a gold standard then there is a critical need for a consensus on the conceptualization of moral decision-making in high-stakes situations and its nomological network, as well as cross-validation of existing measures and potentially development of new measures that capture the agreed-upon conceptualization of moral decision-making. This would pave the way for the development of psychometrically valid tools.

A gold standard measure would need evidence of predictive validity (i.e., predicting real-life outcomes). Predictive validity is not a criterion included in the quality appraisal framework because Terwee et al. (2007) developed the framework to assess the quality of health measures, which are themselves the outcome variable of interest. Therefore, an assessment of what the measures predict is not necessary. The application of a quality appraisal framework is beneficial as it allows for systematic evaluation of measures, and researchers need to be aware of differences in the contexts between the development and application of the framework. However, ultimately a measure of moral decision-making should predict what people actually do, and although predictive validity was not part of our framework we noted its lack in the studies we reviewed.

Ultimately, the ability to validly capture and train moral decision-making in situations where the consequences can involve the life and death of the civilians and combatants is paramount. Our results (see Table 2) show that the included studies either treated moral decision-making as the dependent variable to be predicted (e.g., by age, gender, education level), or validated moral decision-making with measures of other theoretically related constructs (e.g., empathy, cognitive ability, psychopathy). The lack of theoretical and empirical connections between moral decision-making and real-life outcomes invites criticism of how practically meaningful the construct is. Therefore, the predictive validity of moral decision-making measures is an important issue that future studies should address, and it should be a criterion that future reviews consider.

### Limitations

#### Search strategy

The search strategy limited results to only measures of moral decision-making that included life-and-death scenarios and/or items. This decision was based on the goals of our research. We acknowledge that there may be psychometrically sound measures that do not involve life-and-death content. Nevertheless, these measures would contain contexts of great variability such that comparisons between these measures would be difficult. Future studies may conduct systematic reviews of the quality of moral decision-making measures within a specific field (e.g., business, education, sports, engineering).

#### Quality assessment framework, strengths and limitations

The quality appraisal tool used in the present review was originally developed to evaluate self-report measures of officially diagnosable health conditions (Terwee et al., 2007). Our decision to adopt this framework was based on two reasons. First, previous research has successfully applied the framework to non-diagnosable constructs (e.g., Imposter Phenomenon; Mak et al., 2019). Second, the criteria assessed by this framework served as a good guide to evaluating the psychometric properties of measures aimed to capture systematic responses. However, the adoption of the framework placed limitations on our review, such as the lack of focus on predictive validity.

In addition, despite amendments to the quality assessment framework, specific psychometric properties did not necessarily receive higher scores. In certain instances, it was not possible to evaluate certain psychometric properties. For instance, internal consistency estimates of the Process Dissociation Model (Conway and Gawronski, 2013) and the CNI Model (Gawronski et al., 2017) could not be computed because of these measures' design. Rather than taking each dilemma as an item of the measurement, responses from all dilemmas are processed to produce a single score for each factor (e.g., utilitarian and deontological tendencies). Therefore, a rating of “N/A” would imply an inability to evaluate it rather than evidence of poor internal reliability. Another example is the criteria that apply to longitudinal studies. These criteria were designed to evaluate the ability of health measures to detect qualitative changes in health status across time. However, the criteria may be too rigorous for moral decision-making measures, as reporting test-retest reliability would not suffice for a positive rating. Therefore, a lack of a positive rating does not necessarily reflect the poor quality of the measure. For readers, the criteria framework may merely serve as a guide to analyzing the psychometric quality of the measures, whereas the specific findings and statistics may be more informative. Lastly, the criterion “Interpretability”, which refers to the measure's ability to produce qualitative meaning from quantitative scores might make sense in a health/medical setting. However, it may not be applicable in the context of moral decision-making measures. Therefore, ratings on this criterion should bear little weight in evaluating the quality of the measure.

## Conclusions

Overall, the present review extends previous systematic reviews. The results of our review confirm some findings of previous reviews and meta-analyses on moral reasoning (Villegas de Posada and Vargas-Trujillo, 2015; Martí-Vilar et al., 2021) but also highlight novel key findings that are overlooked by past research. Consistent with previous studies, the DIT remains the most used tool to assess moral decision-making. Seven of the twenty included studies used some version of the DIT. However, while measures identified by Martí-Vilar et al. (2021) predominantly relied on self-report responses, most measures identified in the present review used moral dilemmas. This suggests that a substantial amount of research in moral decision-making focuses on aspects of moral decision-making other than moral reasoning. Still, the scope of the moral decision-making construct captured by these measures is very limited. These omissions risk an incomplete and biased understanding of processes in moral decision-making that overestimates the role of cognition while ignoring other processes, such as emotion and metacognition. The present review contributes to the understanding of the current state of research by highlighting this omission and providing a critical foundation for future studies in this domain.

Future research that aims for a gold standard measure of moral decision-making needs to look toward unifying different theories and translating them empirically. This may require the development of new research tools that can be validated in real-world situations. A unifying theory is critical as it would provide a comprehensive taxonomy of different aspects of moral decision-making which are currently overlooked, helping us to develop state-of-the-art knowledge in this critically important area of research.

## Data availability statement

The original contributions presented in the study are included in the article/Supplementary material, further inquiries can be directed to the corresponding author/s.

## Author contributions

BN and KM conducted systematic searches, screened for the inclusion of studies, and evaluated the quality of measures used in studies. BN wrote the manuscript. SK and BB met frequently with BN to provide feedback. All authors also met regularly to review updated drafts and provide feedback. All authors contributed to the article and approved the submitted version.

## Footnotes

^1^Haidt, J., Bjorklund, F., and Murphy, S. (2000). *Moral Dumbfounding: When Intuition Finds No Reason*. University of Virginia (unpublished).
