# Supplementary material for: To kill or not to kill: A systematic literature review of high-stakes moral decision-making measures and their psychometric properties
Source: Front Psychol. 2023 Jan 9;13:1063607. doi: 10.3389/fpsyg.2022.1063607 (PMC9869153; doi:10.3389/fpsyg.2022.1063607)
Supplement: Supplementary file 1 [file Data_Sheet_1.docx]

**Appendix A**

Search Syntax for all databases

| Database | Steps | Search Syntax |
| --- | --- | --- |
| Medline | 1 | exp Decision Making/ |
|  | 2 | exp Morals/ |
|  | 3 | exp Ethics/ |
|  | 4 | 2 or 3 |
|  | 5 | exp Pain Measurement/ |
|  | 6 | moral dilemma.mp. |
|  | 7 | 5 or 6 |
|  | 8 | 1 and 4 |
|  | 9 | moral decision making.mp. [mp=title, abstract, original title, name of substance word, subject heading word, floating sub-heading word, keyword heading word, organism supplementary concept word, protocol supplementary concept word, rare disease supplementary concept word, unique identifier, synonyms] |
|  | 10 | ethical decision making.mp. [mp=title, abstract, original title, name of substance word, subject heading word, floating sub-heading word, keyword heading word, organism supplementary concept word, protocol supplementary concept word, rare disease supplementary concept word, unique identifier, synonyms] |
|  | 11 | moral reasoning.mp. [mp=title, abstract, original title, name of substance word, subject heading word, floating sub-heading word, keyword heading word, organism supplementary concept word, protocol supplementary concept word, rare disease supplementary concept word, unique identifier, synonyms] |
|  | 12 | 8 or 9 or 10 or 11 |
|  | 13 | 7 and 12 |
|  | 14 | limit 13 to ("young adult (19 to 24 years)" or "adult (19 to 44 years)" or "young adult and adult (19-24 and 19-44)" or "middle age (45 to 64 years)" or "middle aged (45 plus years)" or "all aged (65 and over)" or "aged (80 and over)") |
|  | 15 | limit 14 to (english language and humans) |
|  |  |  |
| PsycINFO | 1 | exp Decision Making/ |
|  | 2 | exp Morality/ |
|  | 3 | exp Ethics/ |
|  | 4 | 2 or 3 |
|  | 5 | 1 and 4 |
|  | 6 | moral decision making.mp. [mp=title, abstract, heading word, table of contents, key concepts, original title, tests & measures, mesh] |
|  | 7 | ethical decision making.mp. [mp=title, abstract, heading word, table of contents, key concepts, original title, tests & measures, mesh] |
|  | 8 | moral reasoning.mp. [mp=title, abstract, heading word, table of contents, key concepts, original title, tests & measures, mesh] |
|  | 9 | 5 or 6 or 7 or 8 |
|  | 10 | measurement/ or psychological assessment/ or psychometrics/ or risk assessment/ or screening/ or state trait level measures/ or statistical measurement/ or evaluation/ or test construction/ |
|  | 11 | moral dilemma.mp. [mp=title, abstract, heading word, table of contents, key concepts, original title, tests & measures, mesh] |
|  | 12 | 10 or 11 |
|  | 13 | 9 and 12 |
|  | 14 | limit 13 to human |
|  | 15 | limit 14 to english language |
|  | 16 | limit 15 to "300 adulthood <age 18 yrs and older>" |
|  |  |  |
| Embase | 1 | exp Decision Making/ |
|  | 2 | exp Morals/ |
|  | 3 | exp Ethics/ |
|  | 4 | 2 or 3 |
|  | 5 | exp Pain Measurement/ |
|  | 6 | moral dilemma.mp. |
|  | 7 | 5 or 6 |
|  | 8 | 1 and 4 |
|  | 9 | moral decision making.mp. [mp=title, abstract, original title, name of substance word, subject heading word, floating sub-heading word, keyword heading word, organism supplementary concept word, protocol supplementary concept word, rare disease supplementary concept word, unique identifier, synonyms] |
|  | 10 | ethical decision making.mp. [mp=title, abstract, original title, name of substance word, subject heading word, floating sub-heading word, keyword heading word, organism supplementary concept word, protocol supplementary concept word, rare disease supplementary concept word, unique identifier, synonyms] |
|  | 11 | moral reasoning.mp. [mp=title, abstract, original title, name of substance word, subject heading word, floating sub-heading word, keyword heading word, organism supplementary concept word, protocol supplementary concept word, rare disease supplementary concept word, unique identifier, synonyms] |
|  | 12 | 8 or 9 or 10 or 11 |
|  | 13 | 7 and 12 |
|  | 14 | limit 13 to ("young adult (19 to 24 years)" or "adult (19 to 44 years)" or "young adult and adult (19-24 and 19-44)" or "middle age (45 to 64 years)" or "middle aged (45 plus years)" or "all aged (65 and over)" or "aged (80 and over)") |
|  | 15 | limit 14 to (english language and humans) |
|  |  |  |
| Scopus | | ( TITLE-ABS-KEY ( "moral decision making" OR "ethical decision making" OR "moral reasoning" ) AND TITLE-ABS-KEY ( measurement OR psychometr* OR "moral dilemma" ) ) AND ( LIMIT-TO ( LANGUAGE , "English" ) ) AND ( LIMIT-TO ( EXACTKEYWORD , "Human" ) OR LIMIT-TO ( EXACTKEYWORD , "Humans" ) ) |
|  |  |  |
| Web of Science | | TOPIC: ("ethical decision making" OR "moral decision making" OR "moral reasoning") AND TOPIC: (measurement OR psychometr* OR "moral dilemma") |
|  |  |  |
| Proquest Military Database | | ("ethical decision making" OR "moral decision making" OR "moral reasoning") AND (measurement OR psychometr* OR "moral dilemma") |
